# Draft Genome Sequence of the Moderately Halophilic Methanotroph *Methylohalobius crimeensis* Strain 10Ki

**DOI:** 10.1128/genomeA.00644-15

**Published:** 2015-06-11

**Authors:** Christine E. Sharp, Angela V. Smirnova, Marina G. Kalyuzhnaya, Françoise Bringel, Hisako Hirayama, Mike S. M. Jetten, Valentina N. Khmelenina, Martin G. Klotz, Claudia Knief, Nikos Kyrpides, Huub J. M. Op den Camp, Alexander S. Reshetnikov, Yasuyoshi Sakai, Nicole Shapiro, Yuri A. Trotsenko, Stéphane Vuilleumier, Tanja Woyke, Peter F. Dunfield

**Affiliations:** a Department of Biological Sciences, University of Calgary, Calgary, Alberta, Canada; b Department of Microbiology, Department of Microbiology, San Diego State University, San Diego, California, USA; c Equipe Adaptations et Interactions Microbiennes dans l’Environnement, UMR 7156 UNISTRA–CNRS Génétique Moléculaire, Génomique, Microbiologie, Université de Strasbourg, Strasbourg, France; d Institute of Biogeosciences, Japan Agency for Marine-Earth Science and Technology (JAMSTEC), Yokosuka, Japan; e Department of Microbiology, Radboud University Nijmegen, Nijmegen, the Netherlands; f GK Skryabin Institute of Biochemistry and Physiology of Microorganisms, Russian Academy of Sciences, Pushchino, Russia; g Department of Biological Sciences, University of North Carolina, Charlotte, North Carolina, USA; h Institute of Crop Science and Resource Conservation–Molecular Biology of the Rhizosphere, University of Bonn, Bonn, Germany; i U.S. Department of Energy Joint Genome Institute, Walnut Creek, California, USA; j Division of Applied Life Sciences, Graduate School of Agriculture, Kyoto University, Kyoto, Japan

## Abstract

*Methylohalobius crimeensis* strain 10Ki is a moderately halophilic aerobic methanotroph isolated from a hypersaline lake in the Crimean Peninsula, Ukraine. This organism has the highest salt tolerance of any cultured methanotroph. Here, we present a draft genome sequence of this bacterium.

## GENOME ANNOUNCEMENT

*Methylohalobius crimeensis* strain 10Ki tolerates up to 15% NaCl (1), almost double the level of any other cultured methanotroph (2). The draft genome was sequenced, assembled, and annotated by the U.S. Department of Energy Joint Genome Institute (JGI) using Illumina and Pacific Biosciences (PacBio) technologies. Using the Illumina HiSeq 2000 (3), 20,000,000 reads totaling 1,780 Mb were generated from a long-insert mate pair library and 20,000,000 reads totaling 3,000 Mb from a standard shotgun library. Illumina sequence data were passed through DUK to remove known library preparation and sequencing errors (L. Mingkun, A. Copeland, and J. Han, unpublished data). An SMRTbell library was constructed and sequenced on the PacBio RS platform; 115,902 raw PacBio reads yielded 147,692 adapter-trimmed and quality-filtered subreads totaling 446.8 Mb. Filtered reads comprising 1365.7× Illumina and 127.7× PacBio genome coverage were assembled using AllpathsLG (4). The final draft assembly contained five contigs in five scaffolds. The estimated size of the genome is 3.5 Mbp, with an average G+C content of 58.3%. In total, 3,404 protein-coding genes and 95 pseudogenes were predicted.

Diverse genetic systems for osmotolerance were present, including (i) *ectABCD* genes for ectoine and hydroxyectoine synthesis, along with a second copy of ectoine synthase *ectC*, (ii) a gene encoding a high-affinity importer of choline/glycine betaine driven by a sodium-motive force (5), (iii) three gene copies for choline dehydrogenase and a gene 40% identical to betaine aldehyde dehydrogenase from *Bacillus subtilis*, indicating possible glycine betaine synthesis from choline, and (iv) a pathway for sucrose synthesis and degradation/reutilization, including genes for sucrose-phosphate synthase, sucrose synthase, and fructokinase. Na^+^ export and use of a sodium motive force is suggested by genes encoding a putative Na^+^/H^+^ antiporter localized within an ATP synthase-encoding gene cluster, and a complete *nqr* gene cluster encoding Na^+^-pumping NADH:quinone oxidoreductase (6). There is also a gene cluster for synthesis of gas vesicles, which play a role in adaptation to hypersaline environments (7). Genome comparison with the moderately halophilic methanotroph *Methylomicrobium buryatense* 5G (8) revealed 59.3% overlap of their predicted proteomes (at >60% identity).

The genome did not contain *mmoXYBZDC* genes encoding soluble methane monooxygenase, verifying earlier biochemical tests (1). Two nearly identical and complete operons encoding particulate methane monooxygenase (*pmoCAB*) were detected, along with two other orphan *pmoC* copies. All genes necessary for carbon fixation via the ribulose monophosphate pathway were predicted. Genes encoding a pyrroloquinoline quinone (PQQ)–dependent methanol dehydrogenase, along with an associated cytochrome *c* and other proteins predicted to be involved in Na^2+^-dependent methanol oxidation, were found in an arrangement (*mxaFJGIRSACKLD)* identical to that in *Methyloccoccus capsulatus* (Bath). Tetrahydromethanopterin- and tetrahydrofolate-dependent formaldehyde oxidation pathways and a formate dehydrogenase were encoded. Complete Embden-Meyerhof-Parnas and pentose-phosphate pathways, along with a complete TCA cycle, were predicted, but the Entner-Doudoroff pathway is apparently incomplete. An incomplete serine cycle was predicted due to the absence of phosphoenolpyruvate carboxylase.

Genes encoding for assimilatory nitrate (*nasA*) and nitrite reductase (*nirB*), as well as dissimilatory nitric oxide reductase (*norCB*) and hydroxylamine dehydrogenase (*haoA*), were present. Nitrogen fixation genes were not.

### Nucleotide sequence accession numbers. 

The *Methylohalobius crimeensis* strain 10Ki genome sequence was deposited in GenBank under the accession numbers ATXB01000001 to ATXB01000005.
